# Expression of a mammalian RNA demethylase increases flower number and floral stem branching in *Arabidopsis thaliana*

**DOI:** 10.1002/pld3.70000

**Published:** 2024-08-21

**Authors:** Kasey Markel, Lucas Waldburger, Patrick M. Shih

**Affiliations:** ^1^ Department of Plant and Microbial Biology University of California Berkeley California USA; ^2^ Feedstocks Division Joint BioEnergy Institute Emeryville California USA; ^3^ Environmental Genomics and Systems Biology Division Lawrence Berkeley National Laboratory Berkeley California USA; ^4^ Department of Bioengineering University of California Berkeley California USA; ^5^ Joint BioEnergy Institute Emeryville California USA; ^6^ Biological Systems and Engineering Division Lawrence Berkeley National Laboratory Berkeley California USA; ^7^ Joint Genome Institute Lawrence Berkeley National Laboratory Berkeley California USA; ^8^ Innovative Genomics Institute University of California Berkeley California USA

## Abstract

RNA methylation plays a central regulatory role in plant biology and is a relatively new target for plant improvement efforts. In nearly all cases, perturbation of the RNA methylation machinery results in deleterious phenotypes. However, a recent landmark paper reported that transcriptome‐wide use of the human RNA demethylase FTO substantially increased the yield of rice and potatoes. Here, we have performed the first independent replication of those results and demonstrated broader transferability of the trait, finding increased flower and fruit count in the model species *Arabidopsis thaliana* . We also performed RNA‐seq of our FTO‐transgenic plants, which we analyzed in conjunction with previously published datasets to detect several previously unrecognized patterns in the functional and structural classification of the upregulated and downregulated genes. From these, we present mechanistic hypotheses to explain these surprising results with the goal of spurring more widespread interest in this promising new approach to plant engineering.

## 1 INTRODUCTION

RNA methylation plays a central regulatory role in all eukaryotes and influences RNA processing, translation rate, and transportation (Roundtree et al., 2017). Over 200 types of RNA modification have been identified in plants, of which the most common is N6‐methyladenosine (m^6^A) (Shinde et al., 2023), which is found on approximately .5% of adenosines in mRNA (Luo et al., 2014). Most m^6^A in eukaryotes is found within the consensus motif RRACH (R = A/G, H = A/U/C). Enzymes that add the methyl group to adenosine are often called m^6^A writers, enzymes that remove the methyl group are called m^6^A erasers, and proteins that recognize the methyl group are called m^6^A readers. Many plant traits are known to be affected by m^6^A status, including resistance to fungal pathogens (Guo et al., 2022) and fruit ripening (Zhou et al., 2021). Targeted m^6^A editing has been proposed as a method to improve crop quality, with specific genes proposed to target flowering time and fruit maturation (Zheng et al., 2020).

Recently, Yu et al. (2021) reported that transgenic expression of the mammalian m^6^A eraser FTO substantially increased yield in both potatoes (*Solanum tuberosum*) and rice (*Oryza sativa*). They reported an approximately 50% increase in yield in the field, a remarkably large effect size for a single transgene. In addition to the increase in yield, Yu et al. reported an increase in shoot biomass and polyadenylated mRNA accumulation. The yield increase was explained through an increase in tillering, which suggests reduced apical dominance and increased branching.

FTO overexpression has been shown to increase biomass across a wide range of animal species. FTO has been intensely studied in humans because it is the QTL most strongly associated with obesity (Gao et al., 2010; Merkestein et al., 2015). Overexpression of FTO in mice leads to increased food intake and obesity (Church et al., 2010), whereas *fto* mice display reduced growth and lower body mass (Fischer et al., 2009; Gao et al., 2010). Overexpression of FTO also increases the mitosis rates of mouse fat cells in vitro (Merkestein et al., 2015), and *fto* mutant mice gain less weight even when forced to consume the same amount of food and water as wild‐type mice (Ronkainen et al., 2015). In porcine cell lines, FTO overexpression results in higher accumulation of lipids while *fto* knockouts accumulate lower levels (Wang et al., 2015), and in chicken cells, FTO overexpression increases adipogenesis while FTO silencing reduces adipogenesis (Li et al., 2022).

In contrast, there were no reports prior to Yu et al. that reduction in m^6^A levels results in increased biomass in plants, though many mutants with altered m^6^A levels have been characterized in the last two decades. In *Arabidopsis*, known m^6^A writers include MTA, MTB, FIP37, VIR, FIONA1, and HAKAI. Complete knockout of MTA is embryo‐lethal (Zhong et al., 2008), but plants rescued with embryo‐specific expression display reduced apical dominance and an increase in trichome branch number (Bodi et al., 2012). Silencing of MTA with artificial microRNA results in overproliferation of shoot apical meristems (SAMs) (Shen et al., 2016). Another study found artificial microRNA interference against MTA or VIR resulted in expansion of the SAM onto the petioles of young leaves (Wong et al., 2023). RNA interference against MTB also resulted in a reduction in apical dominance and partial dwarfism (Růžička et al., 2017). *Fip37* knockout lines have reduced apical dominance and severe dwarfism (Růžička et al., 2017), and rescue with embryo‐specific expression resulted in SAM overproliferation (Shen et al., 2016). *Fiona1* mutants have altered RNA splicing patterns (Parker et al., 2022), early flowering, and more branching in the floral stem (Sun et al., 2022). Knockout of the m^6^A eraser ALKBH10B resulted in increased m^6^A, late flowering, and reduced growth, whereas constitutive overexpression resulted in reduced m^6^A and early flowering (Duan et al., 2017). While the details vary depending on which gene is perturbed, it is generally the case that reduction of genome‐wide m^6^A levels in plants results in reduced apical dominance, earlier flowering, and enlarged meristems. Taken together, there are several lines of evidence suggesting how controlling RNA methylation patterns may provide a means to tune agriculturally relevant traits.

Increasing yield potential through genetic improvement is a major goal of plant biology (Woodworth, 1931), and despite massive progress to date, more remains to be accomplished. Given the dramatic nature of the yield increase reported by Yu et al., understanding the transferability of this approach into other plants has been of keen interest. Moreover, understanding how RNA methylation plays a role in this change will help elucidate new avenues in crop improvement. Here, we investigated transgenic expression of FTO in *Arabidopsis* and performed RNA‐seq to generate hypotheses as to the underlying mechanism.

## 2 RESULTS

### 2.1 Overexpression of FTO increases many yield‐associated traits in *Arabidopsis*

**FIGURE 1:**
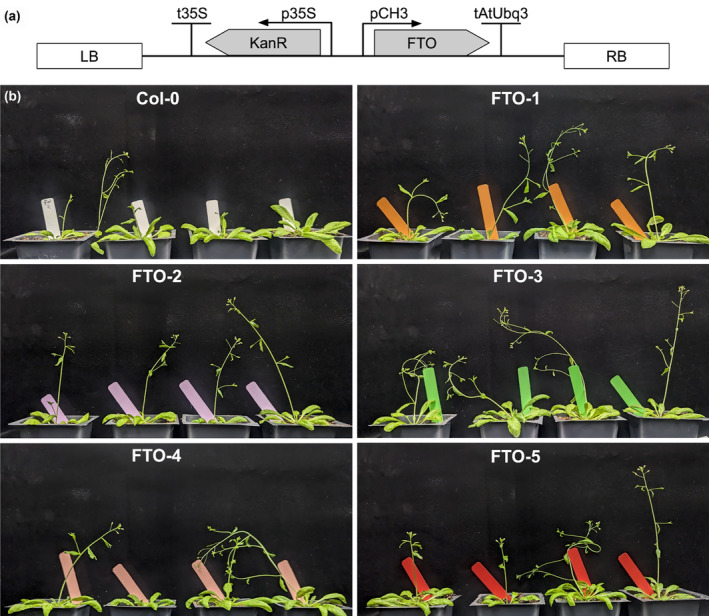
Transgenic expression of FTO causes early bolting in *Arabidopsis*. (a) Genetic design of the synthetic FTO construct. (b) Transgenic plants of the T3 generation. All plants were grown together and randomly selected for imaging on the same day from one of the 10 flats used in this experiment with no cherry picking.

We synthesized an *Arabidopsis* codon‐optimized version of the human FTO gene under the control of the pCH3 promoter (At4G13930), which provides high constitutive expression (Zhou et al., 2023), in a binary plasmid as shown in Figure 1a. We generated *Arabidopsis* stable lines using floral dip and selecting for kanamycin resistance and then grew the plants for two generations and selected for lines with approximately 100% survival on kanamycin, indicating either multiple insertion or homozygosity for FTO. We confirmed that these lines express FTO through RTqPCR (Figure S1, primer sequences and Cq data in Table S1). We grew the T3‐generation FTO plants and found that they bolted earlier than Col‐0 (Figure 1b). Plants were grown an additional 4 days after imaging to allow for maturation and for nearly all plants to bolt, then the plants were destructively phenotyped. Only plants that had bolted (defined as having a floral stem weighing over 100 mg and at least one flower) were included, for a total of 188 plants. Phenotyping consisted of counting the flowers and fruits (siliques), counting the number of shoot tips, and weighing the rosette and floral stems; raw data are available in Table S2.

**FIGURE 2:**
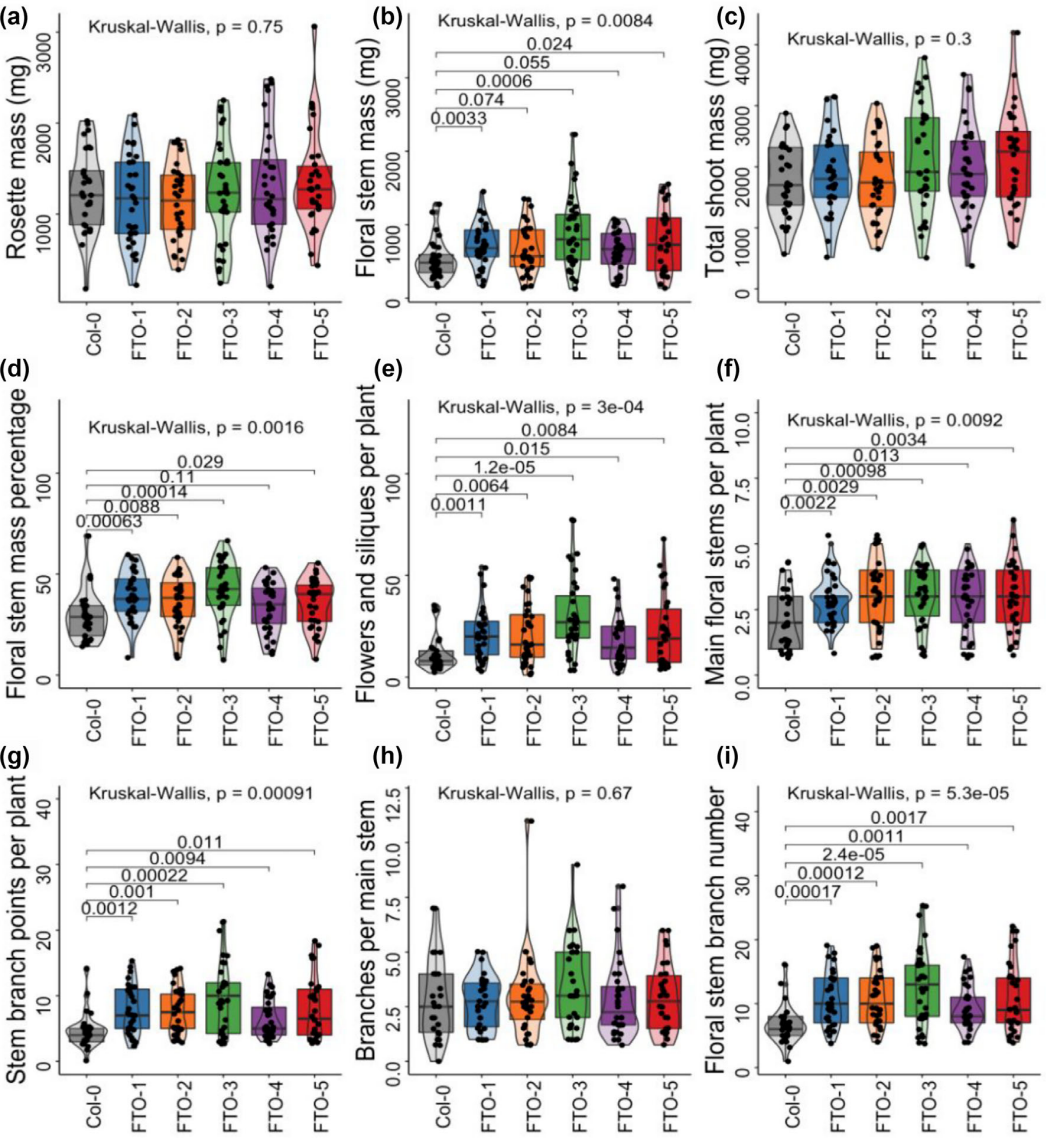
FTO increases many yield‐associated traits in *Arabidopsis*. (a) Rosette mass per plant. (b) Floral stem mass per plant. (c) Total shoot mass per plant. (d) Floral stem mass percentage per plant. (e) Number of flowers and siliques (fruits) per plant. (f) Main floral stems per plant. (g) Stem branch points per plant. (h) Stem branch points per main stem. (i) Floral stem branch number per plant. Boxplot central boxes cover the two central quartiles; points are raw data. Kruskal–Wallis *p*‐value tests for significant difference between any of the groups, if Kruskal–Wallis result was significant, then Wilcoxon test was performed between Col‐0 and all FTO lines with Benjamini–Hochberg correction for multiple comparisons; pairwise *p* values are displayed.

Though Yu et al. claimed a significant increase in stem biomass for both potatoes and rice, we found no difference in *Arabidopsis* rosette mass between FTO plants and Col‐0 wild type (Figure 2a). However, the floral stem mass was significantly increased for all five independent FTO lines (Figure 2b). There was no change in total shoot mass (Figure 2c), suggesting the key difference between FTO and Col‐0 plants was the allocation of growth towards sexual rather than vegetative tissue. Accordingly, there was a significant increase in percentage of stem mass apportioned in floral stem tissue (Figure 2d) for four out of five lines. All FTO lines had a larger number of flowers and siliques than Col‐0, with an average increase of 100% (Figure 2e). Though a priori surprising, this result aligns well with Yu et al.'s finding of an ~200% increase in yield in rice in the greenhouse and ~50% in the field. Our result in a different species in growth chambers is within the range of their observed increases in yield. The effect was statistically significant for all lines, with a maximum *p* value of .015 (Wilcoxon test with Benjamini–Hochberg correction for multiple comparisons).

Since Yu et al. identify an approximately 40% increase in the tiller number as the primary driver of increased yield, we quantified the number of floral stems per plant and the number of floral branch stem points. The total number of floral stem branches increased by an average of 62% (Figure 2f). There was also a significant increase in the number of floral stem branches (Figure 2g), but the number of branches per main floral stem remained constant (Figure 2h). This indicates that the effect was driven by the increase in the number of main stems. The total number of floral stem SAMs—the sum of main stems and stem branch points—also showed a significant increase in all five lines (Figure 2i).

### 2.2 FTO expression alters global gene expression patterns

**FIGURE 3:**
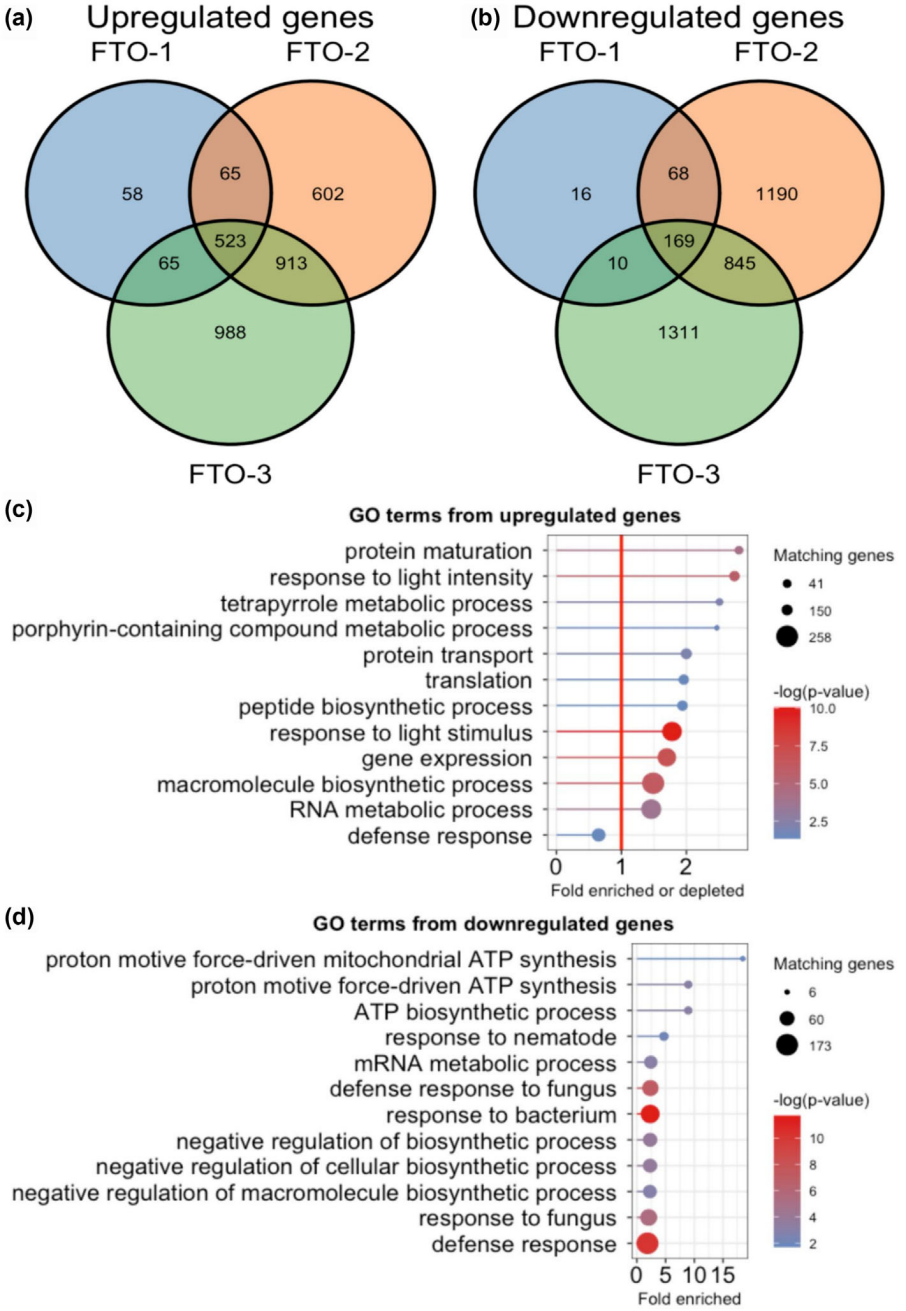
FTO expression alters global gene expression. (a) Venn diagram of significantly upregulated genes in our three transgenic lines compared to Col‐0. Multiple‐comparison adjusted *p*‐value threshold = .01. (b) Venn diagram of significantly downregulated genes in our three transgenic lines compared to Col‐0. Multiple‐comparison adjusted *p*‐value threshold = .01. (c) Lollipop plot of gene ontology (GO) terms enriched among the upregulated genes. Dot size indicates number of genes, line length indicates fold enrichment, and color indicates statistical significance. GO categories with the circle to the right of the red vertical line are enriched; those to the left of the line are depleted. (d) Lollipop plot of GO terms enriched among the downregulated genes. Dot size indicates number of genes, line length indicates fold enrichment, and color indicates statistical significance. All GO categories are enriched, and the lowest enrichment is 1.85×.

We performed RNA‐seq using RNA extracted from seedling shoot tissue to generate mechanistic hypotheses as to why transgenic expression of FTO might have these surprising effects. To ensure robustness of data with respect to experimental and biological variability, we opted for six biological replicates, which enabled us to sequence Col‐0 and three FTO independent lines, 24 samples in total. An average of 34 million reads were mapped per sample, gene‐by‐gene count data are available in Table S3, and raw sequencing data are available through the JGI portal (link in methods). In comparisons to Col‐0, we found FTO‐1 has far fewer differentially expressed genes than FTO‐2 and FTO‐3, both upregulated (Figure 3a) and downregulated (Figure 3b). Interestingly, FTO‐1 also had a smaller phenotypic difference compared to Col‐0 in floral stems per plant (Figure 2f). Given the smaller molecular and phenotypic effects observed for FTO‐1, we decided to focus our analysis on the genes differentially upregulated in either all three lines or FTO‐2 and FTO‐3, leading us to 1,436 upregulated and 1,014 downregulated genes, loci, and additional details of which are included in Table S4.

We performed gene ontology (GO) term enrichment analysis using the PANTHER (Mi et al., 2013) tool through TAIR, with selected GO categories shown in Figure 3c,d for the upregulated and downregulated gene lists, respectively. The full list of significantly enriched or depleted GO categories along with their enrichment, number of genes, and significance can be found in Tables S5 and S6 for upregulated and downregulated gene lists. For the upregulated gene list, the two most enriched categories are “protein maturation” and “response to light intensity.” The next two upregulated categories both relate to porphyrin production, suggesting a change in chlorophyll metabolism. Several growth‐related GO categories were also enriched, such as “peptide biosynthetic process” and “macromolecule biosynthetic process.” “Defense response” is depleted, suggesting the plants are growing at full speed, without regard to potential pathogen attack.

For the downregulated gene list, the most highly enriched GO categories were associated with organellar electron transport chain activity, such as “proton motive force‐driven mitochondrial ATP synthesis” and “ATP biosynthetic process.” Many defense‐related GO categories were enriched among the downregulated genes, such as “response to nematode,” “defense response to fungus,” and “response to bacterium.” Several GO terms for negative regulation of growth such as “negative regulation of biosynthetic process” and “negative regulation of cellular biosynthetic process” were also enriched, suggesting that FTO expression caused the plants to “turn off the brakes” in their growth process. Both the upregulated and downregulated gene lists were enriched for GO terms related to RNA metabolism, such as “RNA metabolic process” and “mRNA metabolic process,” which is not surprising given the alteration to mRNA regulation reported in previous FTO expression papers.

### 2.3 Growth‐associated SAURs are overrepresented among the most highly upregulated genes

We identified the top 100 most upregulated and downregulated genes in each line to gain mechanistic insight into the function of FTO expression relative to Col‐0. Similar to the broader search, we found there was more overlap between FTO‐2 and FTO‐3 and therefore chose to look at genes among the top 100 most‐altered by fold change in either all three lines or just those two. This analysis yielded 41 shared most‐upregulated genes and 36 shared most‐downregulated genes (Table S7). For both the highly upregulated and downregulated genes, there is much more overlap between lines than would be expected by chance (hypergeometric *p* value = 2.53e−76 and 3.79e−64, respectively), confirming that the most highly altered genes among all lines are strongly shared.

Among the 41 highly upregulated genes, four are SAUR genes, which are known to regulate auxin‐induced growth and development (Ren & Gray, 2015). This is approximately 32‐fold higher than expected based on frequency in the genome (hypergeometric *p* value = 7e−06). Overexpression of SAUR genes increases plant growth (Chae et al., 2012; Spartz et al., 2017), and microRNA silencing reduces growth (Chae et al., 2012). Specifically, we found SAUR10, SAUR28, SAUR63, and SAUR68 to be highly upregulated. SAUR10 has been shown to increase growth when overexpressed in *Arabidopsis* (Bemer et al., 2017) and was recently shown to be essential for normal growth and yield in rice (Huang et al., 2023). SAUR28, along with three other nearby SAUR genes, was identified as the largest effect QTL for leaf architecture changes at bolting stage in response to different temperatures (Wang et al., 2019). SAUR63 overexpression has also been shown to increase growth in *Arabidopsis* seedlings (Nagpal et al., 2022). The increased expression of SAUR genes is particularly interesting in light of the reduced apical dominance and increased branching we observed and suggests that auxin regulation may be altered in FTO‐expressing plants.

### 2.4 Organellar genes and repressive transcription factors are overrepresented among the most highly downregulated genes

Among the 36 highly downregulated genes, we found six on the plastid and six on the mitochondrial genome, a ~37‐fold and ~78‐fold overrepresentation, respectively (hypergeometric *p* values = 1.2e−08 and 1.3e−10). In contrast, 0 of the 41 highly upregulated genes were encoded in an organellar genome. Furthermore, among our 1,436 significantly upregulated genes, there were 0 mitochondrial and 0 plastid genes, whereas there were 21 mitochondrial and 34 plastid genes among the 1,014 downregulated genes. This explains the enrichment of three different organellar GO terms—two variants of “proton motive force‐driven ATP synthesis” and “ATP biosynthetic process” among our downregulated genes. Yu et al.'s claim that FTO expression increases nuclear genome‐derived mRNA production offers one possible explanation, because one would expect to see a relative decrease in the abundance of mRNAs derived from organellar genomes. Yu et al. do not report RNA‐seq data for organellar genes, and it should be noted that our library prep process using polyA selection should cause significant depletion of organellar compared to nuclear genome‐derived transcripts, though there is no reason to expect this would differentially affect upregulated versus downregulated genes.

We also noticed several of the highly downregulated genes were transcription factors with known repression activity, including ERF13 (Lv et al., 2021), ERF22 (Nowak et al., 2015), ZAT7 (Ciftci‐Yilmaz et al., 2007), and ZAT10 (Ohta et al., 2001). Interestingly, *erf22* mutants have approximately fivefold increased expression of SAUR10 (Jiang et al., 2023), which concords well with our finding that FTO plants with highly reduced expression of ERF22 also have highly increased expression of SAUR10. Overexpression of ERF13 (Chia, 2013), ZAT7 (Ciftci‐Yilmaz et al., 2007), and ZAT10 (Sakamoto et al., 2004) inhibit growth in *Arabidopsis*, and loss‐of‐function *erf22* mutants (Jiang et al., 2023) and RNAi‐silenced ERF13 (Lee et al., 2010) lines have enhanced growth rates. Taken together, these growth‐reducing genes among the highly downregulated genes suggest a plausible mechanism for the growth‐enhancing effects of FTO. Downregulation of transcriptional repressors is also consistent with the claim by Yu et al. of an overall increase in nuclear‐derived mRNA.

### 2.5 Genes expressed in senescent tissue and genes associated with defense are overrepresented among the genes downregulated by FTO

As another method of exploratory data analysis, we used the Klepikova atlas (Klepikova et al., 2016) through TAIR to identify the tissues in which each gene was most highly expressed. Of the 41 highly upregulated genes, two were most highly expressed in a senescent tissue (senescent leaf petiole and senescent leaf vein). In contrast, 11 of 36 highly downregulated genes were most highly expressed in a senescent tissue (all in senescent leaf petiole), a significant difference (Fisher's exact test two‐tailed *p* value = .0046). Interestingly, all four repressive transcription factors were most highly expressed in senescent leaf petioles. It is possible the downregulation of many genes expressed in senescent tissue is connected to the growth‐enhancing phenotype.

We also collated GO terms for all genes and noticed that 0 of 41 highly upregulated genes and 4 of 36 highly downregulated genes included “pathogen” or “fungus” in their GO terms, a significant difference (Fisher's exact test two‐tailed *p* value = .044). Mutants of one of these genes, AT3‐MMP, have been shown to be more vulnerable to the fungus *Botrytis cinerea* (Zhao et al., 2017). This supports our PANTHER GO‐term analysis, which found “defense response” to be depleted among the upregulated genes (Figure 3c) and found the categories “defense response to bacterium,” “regulation of defense response,” and “defense response to fungus” to all be enriched at least 2.3‐fold among the downregulated genes (Table S6). This reduction in expression level of pathogen‐responsive genes is to be expected in light of long‐established tradeoff between growth and defense (Huot et al., 2014).

### 2.6 Genes upregulated and downregulated by FTO expression differ in sequence properties

**FIGURE 4:**
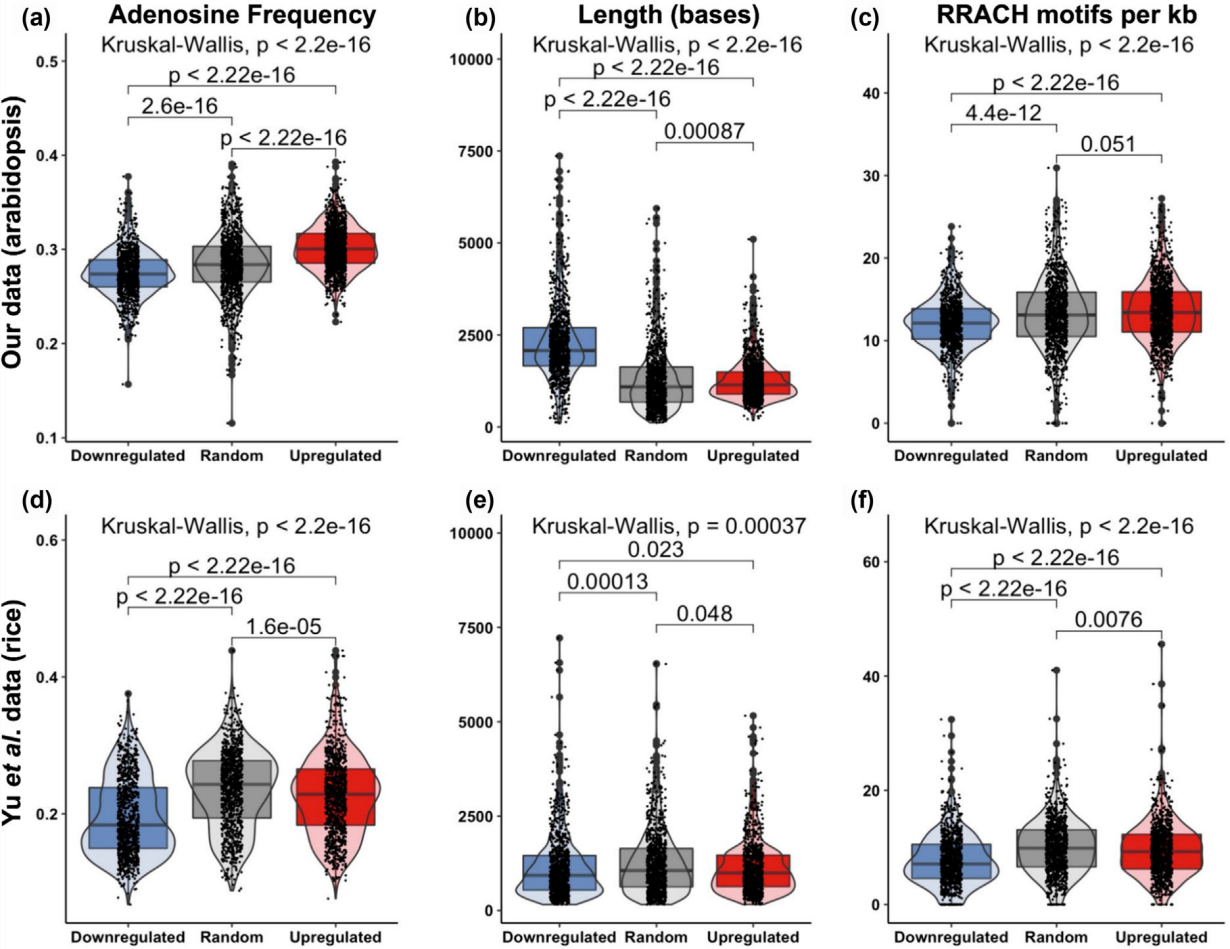
CDS composition of genes upregulated and downregulated by FTO expression are physically distinct. (a) Frequency of adenosine in the CDS of transcripts downregulated and upregulated by FTO expression in our dataset. (c) Length of the CDS of transcripts downregulated and upregulated by FTO expression in our dataset. (c) Frequency of the RRACH (R = A/G, H = A/U/C) motif in the CDS of transcripts downregulated and upregulated by FTO expression in our dataset. (d) Frequency of adenosine in the CDS of transcripts downregulated and upregulated by FTO expression in Yu et al.'s dataset. (e) Length of the CDS of transcripts downregulated and upregulated by FTO expression in Yu et al.'s dataset. (f) Frequency of the RRACH (R = A/G, H = A/U/C) motif in the CDS of transcripts downregulated and upregulated by FTO expression in Yu et al.'s dataset. Boxplot central boxes cover the two central quartiles; points are raw data. Kruskal–Wallis *p*‐value tests for significant difference between any of the groups, if Kruskal–Wallis result was significant, then Wilcoxon test was performed between all three categories of transcript with Benjamini–Hochberg correction for multiple comparisons; pairwise *p* values are displayed.

In general, RNA‐seq data are best understood by considering genes and transcripts as units of biological function, which lends itself to methods of analysis like GO and comparison of differentially expressed genes against other studies of the same gene. However, transcripts are also units of biological structure, and differences in structure such as length or nucleotide composition may be differentially affected by FTO's enzymatic activity acting on RNA. As such, we plotted the average nucleotide frequency within each transcript for three sets of genes: our 1,436 upregulated genes, 1,014 downregulated genes, and a set of 1,200 genes chosen at random from our RNA‐seq counts table as a reference point. The gene lists and sequences are available in Table S4. We found the frequency of adenosine was significantly higher among our upregulated genes compared to both random and downregulated gene lists, with the majority of the gap being between upregulated and the other two categories (Figure 4a). Because m^6^A modification is primarily destabilizing, it stands to reason that with a transcriptome‐wide reduction of m^6^A, we would expect to see adenosine‐rich transcripts more often upregulated.

We next tested whether the length of transcripts differed in length between our three categories and found the downregulated transcripts were much longer than either the random or upregulated transcripts: median length 90% and 81% longer, respectively (Figure 4b). FTO‐mediated removal of m^6^A and destabilization of those transcripts could cause an over‐representation of longer genes among the downregulated gene list. Because m^6^A is known to localize to the motif RRACH (R = A/G, H = A/U/C), we next examined the frequency of that motif within each category of transcripts. Because transcript length differed, we normalized by length and found that downregulated genes had fewer RRACH motifs per kb (Figure 4c). With the naïve assumptions of equal base frequency and random sequence, the RRACH motif occurs about 12 times per kb; among transcribed genes in *Arabidopsis*, we found a slightly higher but comparable frequency. If there is a transcriptome‐wide reduction in a destabilizing epitranscriptomic mark at RRACH motifs, we would expect relative enrichment of transcripts with a high concentration of those motifs among the upregulated genes and a depletion in the downregulated genes, which is exactly what we find. While these sequence‐level differences between upregulated and downregulated genes are robust observations, our interpretations at this point remain speculation.

We also tested the adenosine frequency, length, and RRACH motif concentration in the 5′ and 3′ UTRs and found the differences were either much smaller or nonexistent (Figure S2); it seems to be the CDS in particular that displays these striking sequence‐level differences between our different categories of transcript. These differences also do not seem to be a result of average differences in sequence composition between different functional classes of genes as captured by GO categories—we tested the adenosine frequency, length, and RRACH motif density for all arabidopsis genes of three major GO categories differentially represented in our dataset, “growth,” “response to biological stimulus,” and “response to light.” We found minimal differences in these metrics between GO categories, suggesting the differences found here are due to the peculiarities of the effects of FTO expression, not simply a change in the abundance of genes of a particular GO category (Figure S3).

### 2.7 Trends identified in our dataset are also found in previously‐published RNA‐seq datasets of plants with altered m^6^A deposition

We next investigated whether these structural differences between downregulated, randomly selected, and upregulated genes were present in previous RNA‐seq datasets of m^6^A‐altered plants. We begin with Yu et al.'s rice shoot RNA‐seq dataset, which we used to generate even‐sized groups of downregulated, randomly selected, and upregulated genes in the FTO plants. We found genes upregulated by FTO to have higher CDS adenosine frequency (Figure 4d), matching our results and suggesting commonalities in the mechanism of action between rice and *Arabidopsis*. Median CDS length is only negligibly different between upregulated and downregulated genes in their dataset, though much like our dataset, the upregulated gene list had fewer very long genes (Figure 4e), which is not an artifact of different size gene sets since our gene lists are numerically balanced. Similar to our dataset, downregulated genes were depleted for RRACH motifs compared to randomly‐selected or upregulated genes (Figure 4f). We performed GO analysis using PANTHER on the upregulated and downregulated gene lists, with selected GO terms plotted in Figure S4 and full lists available as Tables S8 and S9, respectively. Genes associated with response to light intensity are over‐represented among their upregulated gene list, including “response to high light intensity,” “response to light intensity,” and “response to red or far red light,” matching findings from our data (Figure S4 and Table S8). Genes related to protein synthesis were also over‐represented, including “peptide metabolic process” and “translation,” also matching our results. Among the downregulated genes, the most enriched GO term was “response to chitin,” suggesting FTO‐expressing plants are tuning down their defenses against fungi or arthropods. “Defense response,” “regulation of defense response,” “interspecies interaction,” “response to biotic stimulus,” “defense response to other organism,” and “response to fungus” are all overrepresented among their downregulated gene lists, matching our results (Figure S4 and Table S9).

To better understand how our results match previous *Arabidopsis* data, we also analyzed a publicly available RNA‐seq dataset from *fiona1* mutant plants (Parker et al., 2022). We reason that mutants of an m^6^A writer may behave similarly to our m^6^A eraser overexpression lines. We chose mutants of this specific m^6^A writer because the mutants of most others have severe phenotypes, whereas *fiona1* plants grow to maturity and obtain comparable size to wild‐type plants. We performed GO analysis using PANTHER after identifying upregulated and downregulated genes between Col‐0 and *fiona1* plants, selected GO terms of which are plotted in Figure S5 and full results of which are available as Tables S10 and S11, respectively. GO terms “response to high light intensity” and “response to light” were enriched among the upregulated genes, matching results from our data and our analysis of Yu et al.'s data. We found “response to biotic stimulus,” “defense response to other organism,” and “defense response” were all enriched at least 1.85‐fold among the downregulated genes, whereas no defense categories were either depleted among the downregulated genes or enriched among the upregulated genes (Figure S4 and Table S11). The organellar genes were also non‐evenly distributed between upregulated and downregulated genes in *fiona1*; among the upregulated genes, there were 10 plastid and 1 mitochondrial gene, whereas among the downregulated genes were 40 mitochondrial and 1 plastid gene. The strong enrichment of mitochondrial genes in the downregulated gene list matches our findings with FTO, whereas the distribution of plastid genes is opposite to our finding.

We investigated several additional published RNA‐seq datasets specifically for the question of organellar genes being overrepresented among the downregulated genes, and did not find a clear pattern—microRNA knockdown of the m^6^A writer MTA (Wong et al., 2023) revealed in an overrepresentation of mitochondrial genes among the downregulated genes, but plastid genes were overrepresented among upregulated genes, matching the *fiona1* results. Mutants of the m^6^A writer FIP37 (Wong et al., 2023) show an enrichment for plastid genes among upregulated genes, whereas mitochondrial genes show no enrichment. Mutant plants deficient for the native m^6^A eraser ALKBH10B also show neither enrichment nor depletion for plastid or mitochondrial genes (Duan et al., 2017).

## 3 DISCUSSION

Our findings broadly support the findings of Yu et al., demonstrating how the FTO overexpression approach may be a broadly transferable trait and should encourage future research using modulation of RNA methylation status as a tool to improve plant yield. We find a significant increase in flower and fruit number, stem branching, and reproductive stem biomass. We present several potential hypotheses for the effect, such as the upregulation of SAUR genes and repression of repressive transcription factors. We also present two striking observations for further investigation: an inconsistent but strong pattern of disproportional representation of organellar genes among the lists upregulated and downregulated by FTO, and differences between upregulated and downregulated gene sets such as a reduction in adenosine frequency and RRACH motif abundance among downregulated genes. We believe our data in conjunction with that of Yu et al. are sufficient to warrant more widespread research into the effects of FTO expression on plant yield and present directions for future research.

Public opinion has been a major barrier to the widespread deployment of transgenic crops, and we recognize that the introduction of a transgene derived from humans into crops represents a significant public relations liability. To hedge these concerns, future studies pursuing FTO overexpression may benefit from avoiding the human version of the gene and instead utilize orthologs from close mammalian relatives such as the porcine or bonobo orthologs, which are particularly similar in amino acid sequence. It may also be possible to achieve similar growth improvements with a transgene‐free approach by reducing the level of RNA methylation through selective reduction of m^6^A writers rather than the addition of a phylogenetically distant m^6^A eraser. However, the growth defects in reported m^6^A writer mutants suggest this will need to be approached carefully and will likely require more mechanistic understanding of the basis of the growth enhancement phenotype. Fortunately, new sequencing technologies are rapidly improving our ability to detect RNA modification (Wang & Jia, 2020), which may light the way towards knowing which RNA species in which tissues at which developmental stages are involved in this remarkable growth‐enhancing phenotype.

## 4 METHODS

### 4.1 Plant growth

Col‐0 *Arabidopsis* were grown to flowering stage for transformation by floral dip and transformed with *Agrobacterium tumefaciens* strain GV3101 following standard methods, and the T0 progeny seeds selected for resistance to 50 mg/L kanamycin during axenic growth on agar plates. Over 50 independent lines were obtained, which were selected and propagated to increase seed and prepare for the main growth experiments with five lines of homozygous T3 plants. These plants were grown in an AR‐95L3 chamber designed for *Arabidopsis* (Percival Scientific) under long day conditions with 12:12 light:dark, 25°C, 60% relative humidity, “Professional Growing Mix” soil (Sungrow Horticulture) with Peter's Professional 20‐20‐20 (ICL Growing Solutions) diluted into the water at 1 g/L once per week. Plants were kept well watered in standard 1,020 flats with 24 pots per flat.

### 4.2 Accession numbers

Sequence data from this article can be found in the EMBL/GenBank database or the *Arabidopsis* Genome Initiative database under the following accession numbers: the human FTO enzyme used in this study is Genbank: KAI2578515, the porcine ortholog is GenBank: ADD69819. MTA is At4g10760, MTB is At4g09980, FIP37 is At3g54170, VIR is At3G05680, FIONA1 is AtMG00980, HAKAI is At5G01160, ECT2 is At3G13460, ECT3 is At5G61020, ECT4 is At1G55500, CPSF30 is At1G30460, and ALKBH10B is At4G02940. SAUR28 is At3G03830, and SAUR10 is At2G18010. All other gene names are available in Table S4. Raw sequencing data are available through the JGI portal (https://genome.jgi.doe.gov/portal/RNAofexinplants/RNAofexinplants.info.html).

### 4.3 RNA extraction and library preparation

From 7‐day‐old seedlings grown under axenic conditions on 1/2 MS plates, 15–30 mg of shoot tissue was excised and immediately flash‐frozen in liquid nitrogen and stored at −80°C. RNA extraction was performed using E.Z.N.A Total RNA kit (Omega BioTek) following manufacturer directions. Extracted RNA was then DNAse treated with TURBO DNA‐Free kit (Thermo Scientific). mRNA was isolated from an input of 1 μg of total RNA with oligo dT magnetic beads and fragmented to 300–400 bp with divalent cations at a high temperature. Using TruSeq stranded mRNA kit (Illumina), the fragmented mRNA was reverse transcribed to create the first strand of cDNA with random hexamers and SuperScript™ II Reverse Transcriptase (Thermo Fisher Scientific) followed by second strand synthesis. The double‐stranded cDNA fragments were treated with A‐tailing, ligation with JGI's unique dual indexed adapters (IDT), and enriched using eight cycles of PCR. The prepared libraries were quantified using KAPA Biosystems' next‐generation sequencing library qPCR kit and run on a Roche LightCycler 480 real‐time PCR instrument. Sequencing of the flowcell was performed on the Illumina NovaSeq sequencer using NovaSeq XP V1.5 reagent kits, S4 flowcell, following a 2 × 151 indexed run recipe.

### 4.4 RTqPCR

Total mRNA was extracted using E.Z.N.A. plant RNA kit (Omega Bio‐tek) following manufacturer directions using the RB lysis buffer variation and on‐column DNase digestion; cDNA synthesis was achieved with SSIV Vilo IV kit using random hexamers (Thermo Fisher). Quantitative PCR was performed using a CFX96 Real‐Time thermocycler (Bio‐Rad) programmed for detection of SYBR intercalating dye with the following temperature programming: 95°C for 3 min, then 95°C for 30 s, 60°C for 45 s repeated 38 times, and then a gradual increase from 65°C to 95°C at .5°C/min to generate melt curves. Sso‐Advanced Universal SYBR Green Supermix (Bio‐Rad) was used for qPCR amplification. A previously validated primer set was used to amplify EF1α for internal normalization, three sets of primers for FTO were designed with Benchling's qPCR primer design wizard, and the best was selected on the basis of consistent amplification of template DNA; primer sequences and raw data are available in Table S1. Melt curves for the product of all primer sets were unimodal and steep, suggesting only a single product was formed for each primer set. No reverse‐transcriptase controls showed no amplification within the dynamic range of samples, confirming the efficacy of DNAse treatment, and no template controls instituted at the beginning of RNA extraction with no plant matter and kept in parallel with real samples throughout all molecular steps did not amplify, confirming lack of contamination with extraneous DNA. Normalized relative expression was calculated using the delta‐delta‐Cq method. Since no amplification for the FTO primers was found in the Col‐0 samples, a default CQ value of 38 was input and used as an upper bound on the potential amplification of the FTO primers in the Col‐0 samples. In this way, we could express the enrichment of FTO amplification of the transgenic lines and compare it to a “limit of detection” in the control, which we graph as normalized to 1. This method should underestimate the relative enrichment of FTO in the FTO‐transgenic lines compared to Col‐0, but enables comparison of the expression levels between lines.

### 4.5 RNA‐seq data analysis

#### 4.5.1 Read preprocessing

Raw fastq file reads were filtered and trimmed using the JGI QC pipeline resulting in the filtered fastq file (*.filter‐RNA.gz files). Using BBDuk, raw reads were evaluated for artifact sequence by kmer matching (kmer = 25), allowing one mismatch, and detected artifact was trimmed from the 3′ end of the reads. RNA spike‐in reads, PhiX reads and reads containing any Ns were removed. Quality trimming was performed using the phred trimming method set at Q6. Finally, following trimming, reads under the length threshold were removed (minimum length 25 bases or 1/3 of the original read length—whichever is longer).

#### 4.5.2 Read alignment and counting

Filtered reads from each library were aligned to the reference genome using HISAT2 version 2.2.1 (with −k 1 flag) (BAMs/ directory). Strand‐specific coverage bigWig files (fwd and rev) were generated using deepTools v3.1(bigWigs/directory). featureCounts was used to generate the raw gene counts (counts.txt) file using gff3 annotations. Only primary hits assigned to the reverse strand were included in the raw gene counts (−s 2 −p—primary options). Raw gene counts were used to evaluate the level of correlation between biological replicates using Pearson's correlation and determine which replicates would be used in the DGE analysis (replicate_analysis.txt, replicate_analysis_heatmap.pdf). In the heatmap view, the libraries were ordered as groups of replicates. The cells containing the correlations between replicates have a purple (or white) border around them. FPKM and TPM normalized gene counts are also provided (fpkm_counts.txt and tpm_counts.txt).

#### 4.5.3 Strandedness

Features assigned to the forward strand were also tabulated (−s 1 −p—primary options). Strandedness of each library was estimated by calculating the percentage of reverse‐assigned fragments to the total assigned fragments (reverse plus forward hits).

#### 4.5.4 Differential gene expression

DESeq2 (version 1.30.0) was used to determine which genes were differentially expressed between pairs of conditions. The parameters used to call a gene differentially expressed between conditions were adjusted *p* value < .01. The file DGE_summary.txt includes the log2 fold change, adjusted Pval, and whether the gene is significantly differentially expressed (TRUE/FALSE/NA) for each pair of conditions specified. We also include shrunken log2 fold changes from DESeq2's “normal” method for visualization and ranking. Refer to the sample summary table above to match SampleName with the ConditionNumber assigned for analysis. Individual results for each pairwise comparison are in the directory pairwise DGE results. Note: Raw gene counts (counts.txt), not normalized counts, are used for DGE analysis. DESeq2 conducts its own internal normalization using a sophisticated sampling model.

#### 4.5.5 Gene set enrichment analysis

Gene set enrichment analysis evaluates whether certain gene sets or pathways contain more differentially expressed genes than expected by chance. If KEGG annotations were available for the genes in this reference genome, PADOG (version 1.36.0) was used to calculate gene set enrichment of the KEGG pathways found in the genome. Gene annotations are summarized in gene_functional_annotations.tsv, and KEGG pathways with at least three genes present in the reference genome are provided in KEGG_gene_sets.tsv. Figures were produced using RStudio with the Tidyverse package (Wickham et al., 2019).

#### 4.5.6 Analysis of Yu et al. dataset

We used the shoot standard RNA‐seq (as opposed to m^6^A‐seq) data and sorted genes by average fold‐change between wild‐type and FTO plants. We then generated our three gene lists by selecting the 1,000 most‐upregulated genes, 1,000 most‐downregulated genes, and 1,000 genes selected using a random number generator.

## AUTHOR CONTRIBUTIONS

Kasey Markel conceived and performed the experiments and wrote the manuscript. Lucas Waldburger assisted with bioinformatic analysis. Patrick M. Shih supervised and provided funding. All authors have read and approved the manuscript.

## CONFLICT OF INTEREST STATEMENT

The authors declare no competing interests.

## PEER REVIEW

The peer review history for this article is available in the supporting information for this article.

## Supporting information


**Table S1:** primers used for RTqPCR and RTqPCR raw data.

**Table S2:** Phenotypes of T3 generation plants.

**Table S3:** RNA‐seq counts table.

**Table S4:** Gene IDs, sequence, and other parameters for upregulated, downregulated, and randomly‐selected gene lists.

**Table S5:** PANTHER GO Term analysis of upregulated genes.
**Table S6:** PANTHER GO Term analysis of downregulated genes.
**Table S7:** Highly differentially expressed genes.
**Table S8:** PANTHER GO Term analysis of upregulated genes from Yu et al.'s dataset.
**Table S9:** PANTHER GO Term analysis of downregulated genes from Yu et al.'s dataset.
**Table S10:** PANTHER GO Term analysis of upregulated genes from publicly‐available *fiona1* dataset.
**Table S11:** PANTHER GO Term analysis of downregulated genes from publicly‐available *fiona1* dataset.

**Data S1** Supporting Information.

**Figure S1:** **FTO is expressed in all transgenic lines used.** RTqPCR normalized relative expression data for all lines. Since the FTO primers did not amplify in the Col‐0 lines, we input a Cq of our maximum cycle number for normalization as an “upper bound”, and graphed with that normalized to 1. Note the logarithmic scale on the Y axis. Data and primer sequences are available in Supplemental Table 1.
**Supplemental Figure 2: Sequence composition differences of UTRs of genes upregulated and downregulated by FTO expression are smaller than those of CDSs.** A) Frequency of adenosine in the 5’ UTR of transcripts downregulated and upregulated by FTO expression. B) Frequency of adenosine in the CDS of transcripts downregulated and upregulated by FTO expression. C) Frequency of adenosine in the 3’ UTR of transcripts downregulated and upregulated by FTO expression. D) Length of the 5’ UTR of transcripts downregulated and upregulated by FTO expression. E) Length of the CDS of transcripts downregulated and upregulated by FTO expression. F) Length of the 3’ UTR of transcripts downregulated and upregulated by FTO expression. G) RRACH motifs per kb in the 5’ UTR of transcripts downregulated and upregulated by FTO expression. H) RRACH motifs per kb in the CDS of transcripts downregulated and upregulated by FTO expression. I) RRACH motifs per kb in the 3’ UTR of transcripts downregulated and upregulated by FTO expression. Boxplot central boxes cover the two central quartiles, points are raw data. Kruskal‐Wallis p‐value tests for significant difference between any of the groups, if Kruskal‐Wallis result was significant then Wilcoxon test was performed between all three categories of transcript with Benjamini‐Hochberg correction for multiple comparisons, pairwise p‐values displayed.
**Supplemental Figure 3: Sequence‐level differences between GO categories are minimal**. A) Adenosine frequency of genes for three GO terms. B) Length of genes for three GO terms. C) RRACH motif abundance per kb for three GO terms. All panels include the CDS of all genes annotated as within the GO category on TAIR. Boxplot central boxes cover the two central quartiles, points are raw data. Kruskal‐Wallis p‐value tests for significant difference between any of the groups, if Kruskal‐Wallis result was significant then Wilcoxon test was performed between all three categories of transcript with Benjamini‐Hochberg correction for multiple comparisons, pairwise p‐values displayed.
**Supplemental Figure 4: GO analysis of Yu et al's FTO rice RNA‐seq reveals many of the same trends identified in our data.** A) Lollipop plot of selected GO terms enriched among Yu et al's FTO rice upregulated genes. Dot size indicates number of genes, line length indicates fold enrichment, color indicates statistical significance. GO categories with the circle to the right of the red vertical line are enriched, those to the left of the line are depleted. B) Lollipop plot of selected GO terms enriched among Yu et al's FTO rice downregulated genes. Dot size indicates number of genes, line length indicates fold enrichment, color indicates statistical significance. GO categories with the circle to the right of the red vertical line are enriched, those to the left of the line are depleted. Full lists of GO categories and associated data are available in Supplemental Table 7 and Supplemental Table 8 for the upregulated and downregulated gene lists respectively.
**Supplemental Figure 5: GO analysis of published f*iona1* mutant data reveals many of the same trends identified in our data.** A) Lollipop plot of selected Gene Ontology (GO) terms enriched among *fiona1* upregulated genes. Dot size indicates number of genes, line length indicates fold enrichment, color indicates statistical significance. All GO categories displayed are enriched, the lowest enrichment is 2.91x. B) Lollipop plot of selected GO terms enriched among *fiona1* downregulated genes. Dot size indicates number of genes, line length indicates fold enrichment, color indicates statistical significance. GO categories with the circle to the right of the red vertical line are enriched, those to the left of the line are depleted. Full lists of GO categories and associated data are available in Supplemental Table 9 and Supplemental Table 10 for the upregulated and downregulated gene lists respectively.

## Data Availability

All data generated for this manuscript are available in the manuscript, supplementary tables and figures, publicly available code, and JGI and JBEI public databases linked in the manuscript.
