# Supplementary material for: Expression of a mammalian RNA demethylase increases flower number and floral stem branching in Arabidopsis thaliana
Source: Plant Direct. 2024 Aug 21;8(8):e70000. doi: 10.1002/pld3.70000 (PMC11636547; doi:10.1002/pld3.70000)
Supplement: Supplementary file 7 — Figure S1: FTO is expressed in all transgenic lines used. RTqPCR normalized relative expression data for all lines. Since the FTO primers did not amplify in the Col‐0 lines, we input a Cq of our maximum cycle number for normalization as an “upper bound”, and graphed with that normalized to 1. Note the logarithmic scale on the Y axis. Data and primer sequences are available in Supplemental Table 1. Supplemental Figure 2: Sequence composition differences of UTRs of genes upregulated and downregulated by FTO expression are smaller than those of CDSs. A) Frequency of adenosine in the 5’ UTR of transcripts downregulated and upregulated by FTO expression. B) Frequency of adenosine in the CDS of transcripts downregulated and upregulated by FTO expression. C) Frequency of adenosine in the 3’ UTR of transcripts downregulated and upregulated by FTO expression. D) Length of the 5’ UTR of transcripts downregulated and upregulated by FTO expression. E) Length of the CDS of transcripts downregulated and upregulated by FTO expression. F) Length of the 3’ UTR of transcripts downregulated and upregulated by FTO expression. G) RRACH motifs per kb in the 5’ UTR of transcripts downregulated and upregulated by FTO expression. H) RRACH motifs per kb in the CDS of transcripts downregulated and upregulated by FTO expression. I) RRACH motifs per kb in the 3’ UTR of transcripts downregulated and upregulated by FTO expression. Boxplot central boxes cover the two central quartiles, points are raw data. Kruskal‐Wallis p‐value tests for significant difference between any of the groups, if Kruskal‐Wallis result was significant then Wilcoxon test was performed between all three categories of transcript with Benjamini‐Hochberg correction for multiple comparisons, pairwise p‐values displayed. Supplemental Figure 3: Sequence‐level differences between GO categories are minimal. A) Adenosine frequency of genes for three GO terms. B) Length of genes for three GO terms. C) RRACH motif abundance per kb fo [file PLD3-8-e70000-s006.docx]

**Supplemental Figures**

**
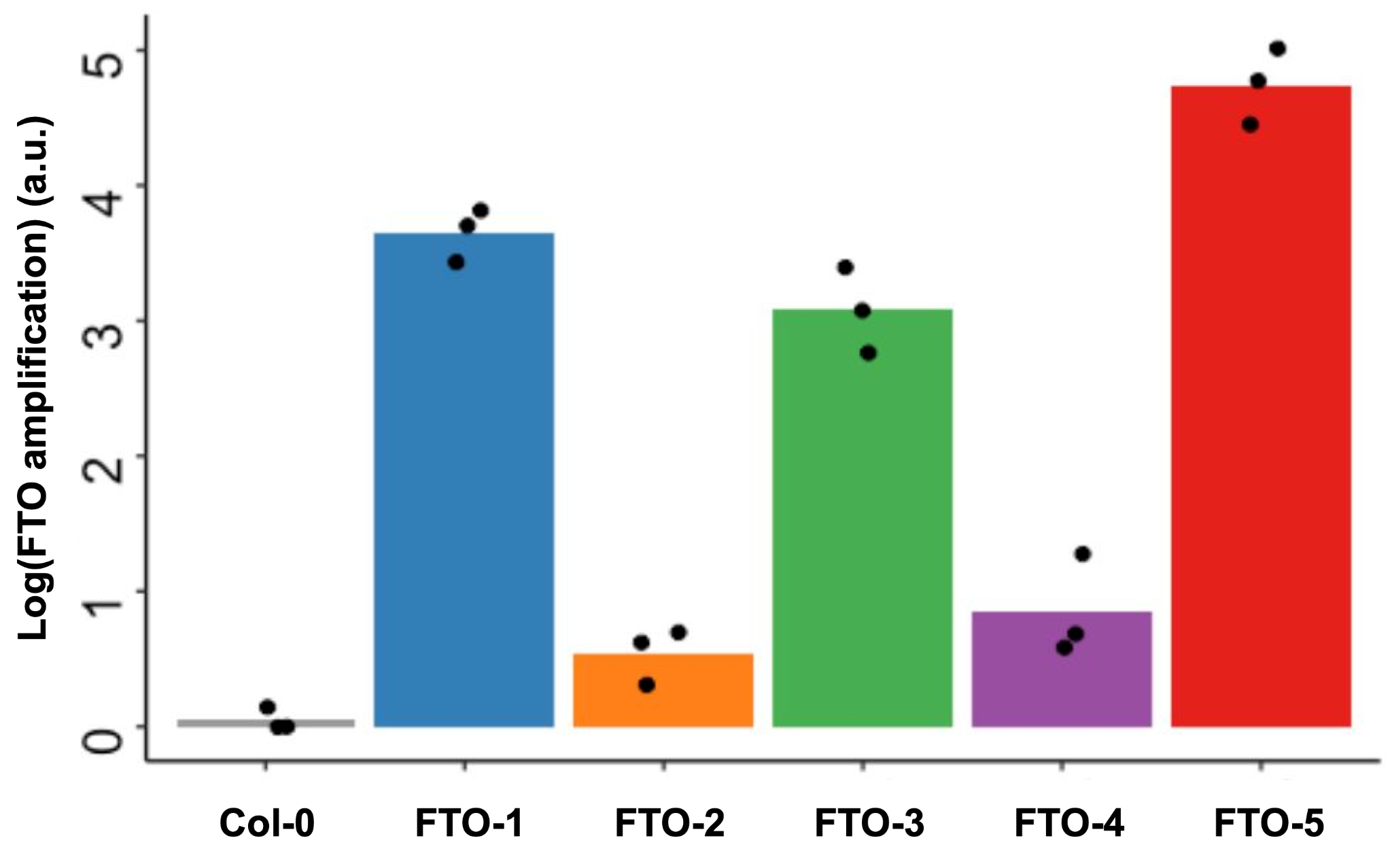
**

**Supplemental Figure 1: FTO is expressed in all transgenic lines used.** RTqPCR normalized relative expression data for all lines. Since the FTO primers didn’t amplify in the Col-0 lines, we input a Cq of our maximum cycle number for normalization as an “upper bound”, and graphed with that normalized to 1. Note the logarithmic scale on the Y axis. Data and primer sequences are available in Supplemental Table 1.

**
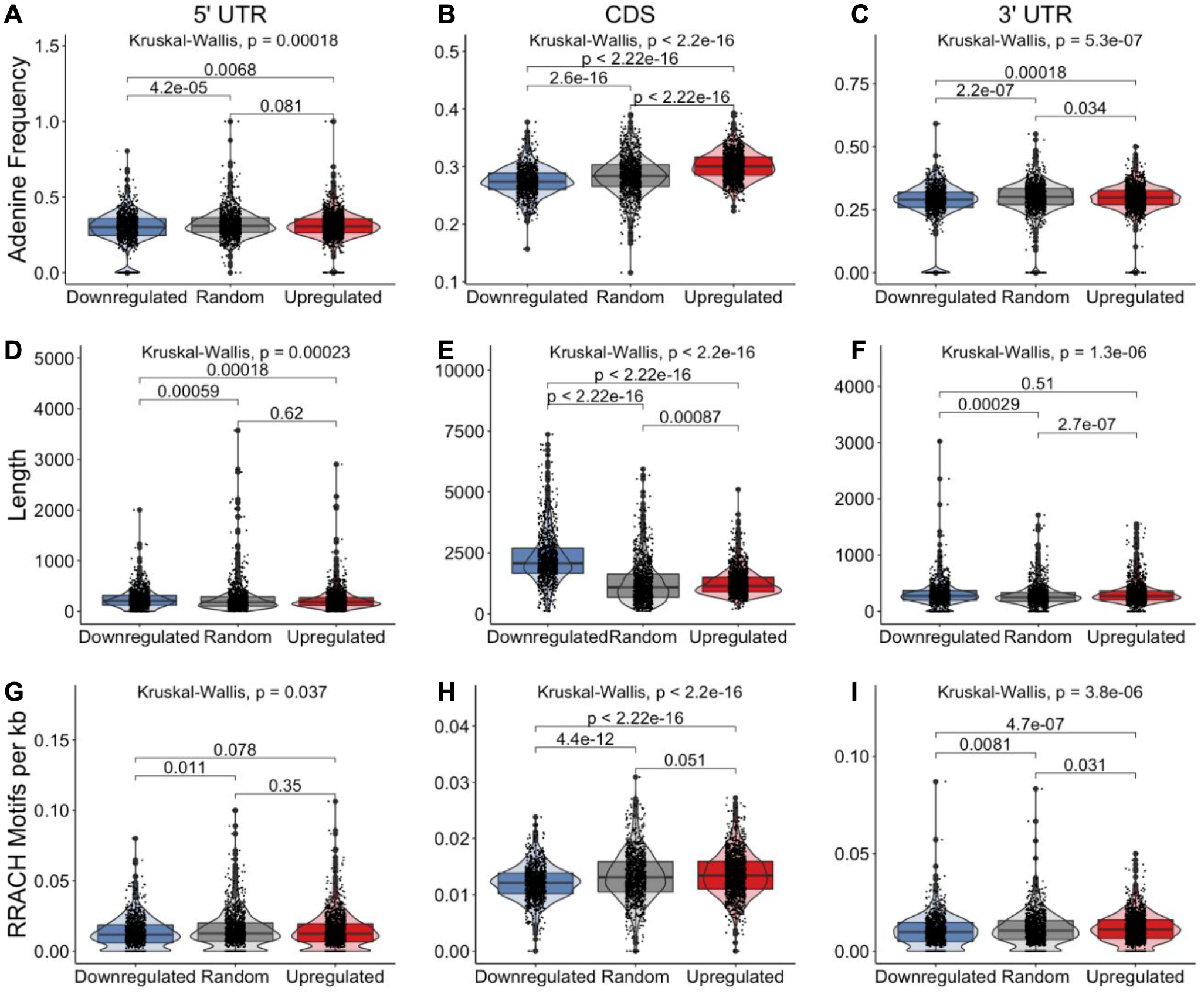
**

**Supplemental Figure 2: Sequence composition differences of UTRs of genes upregulated and downregulated by FTO expression are smaller than those of CDSs.** A) Frequency of adenosine in the 5’ UTR of transcripts downregulated and upregulated by FTO expression. B) Frequency of adenosine in the CDS of transcripts downregulated and upregulated by FTO expression. C) Frequency of adenosine in the 3’ UTR of transcripts downregulated and upregulated by FTO expression. D) Length of the 5’ UTR of transcripts downregulated and upregulated by FTO expression. E) Length of the CDS of transcripts downregulated and upregulated by FTO expression. F) Length of the 3’ UTR of transcripts downregulated and upregulated by FTO expression. G) RRACH motifs per kb in the 5’ UTR of transcripts downregulated and upregulated by FTO expression. H) RRACH motifs per kb in the CDS of transcripts downregulated and upregulated by FTO expression. I) RRACH motifs per kb in the 3’ UTR of transcripts downregulated and upregulated by FTO expression. Boxplot central boxes cover the two central quartiles, points are raw data. Kruskal-Wallis p-value tests for significant difference between any of the groups, if Kruskal-Wallis result was significant then Wilcoxon test was performed between all three categories of transcript with Benjamini-Hochberg correction for multiple comparisons, pairwise p-values displayed.


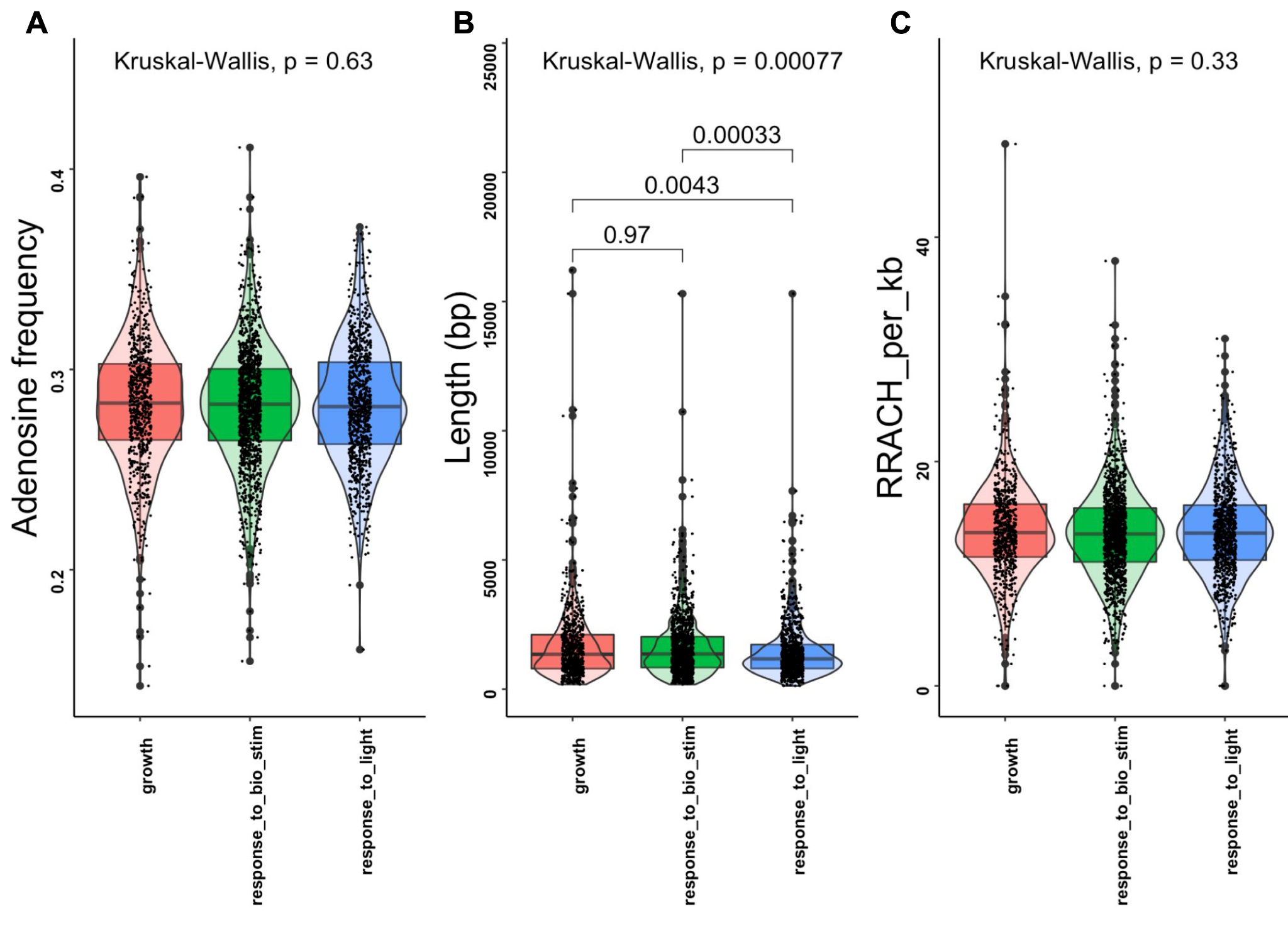


**Supplemental Figure 3: Sequence-level differences between GO categories are minimal**. A) Adenosine frequency of genes for three GO terms. B) Length of genes for three GO terms. C) RRACH motif abundance per kb for three GO terms. All panels include the CDS of all genes annotated as within the GO category on TAIR. Boxplot central boxes cover the two central quartiles, points are raw data. Kruskal-Wallis p-value tests for significant difference between any of the groups, if Kruskal-Wallis result was significant then Wilcoxon test was performed between all three categories of transcript with Benjamini-Hochberg correction for multiple comparisons, pairwise p-values displayed.

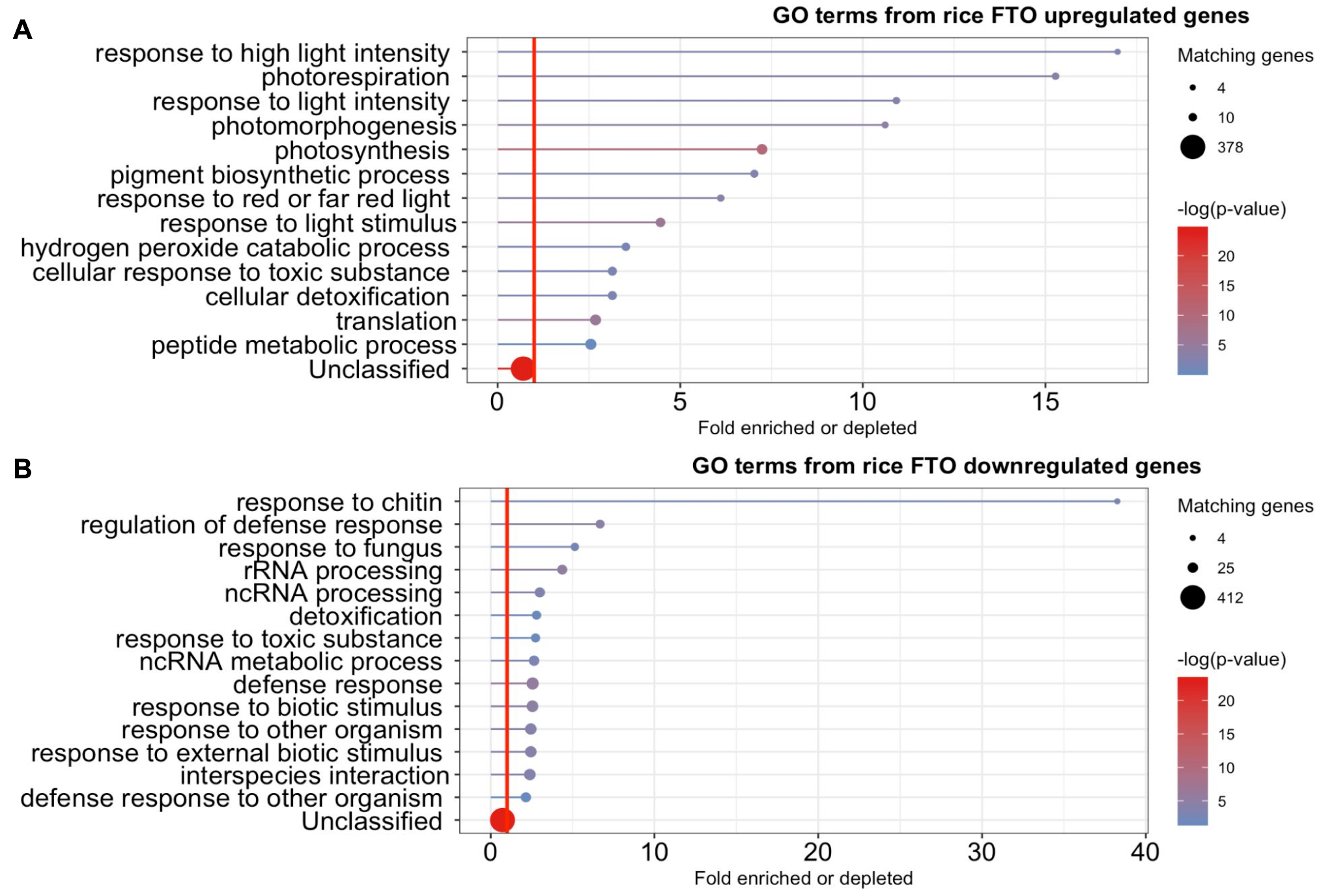

**Supplemental Figure 4: GO analysis of Yu *et al*’s FTO rice RNA-seq reveals many of the same trends identified in our data.** A) Lollipop plot of selected GO terms enriched among Yu *et al*’s FTO rice upregulated genes. Dot size indicates number of genes, line length indicates fold enrichment, color indicates statistical significance. GO categories with the circle to the right of the red vertical line are enriched, those to the left of the line are depleted. B) Lollipop plot of selected GO terms enriched among Yu *et al*’s FTO rice downregulated genes. Dot size indicates number of genes, line length indicates fold enrichment, color indicates statistical significance. GO categories with the circle to the right of the red vertical line are enriched, those to the left of the line are depleted. Full lists of GO categories and associated data are available in Supplemental Table 7 and Supplemental Table 8 for the upregulated and downregulated gene lists respectively.


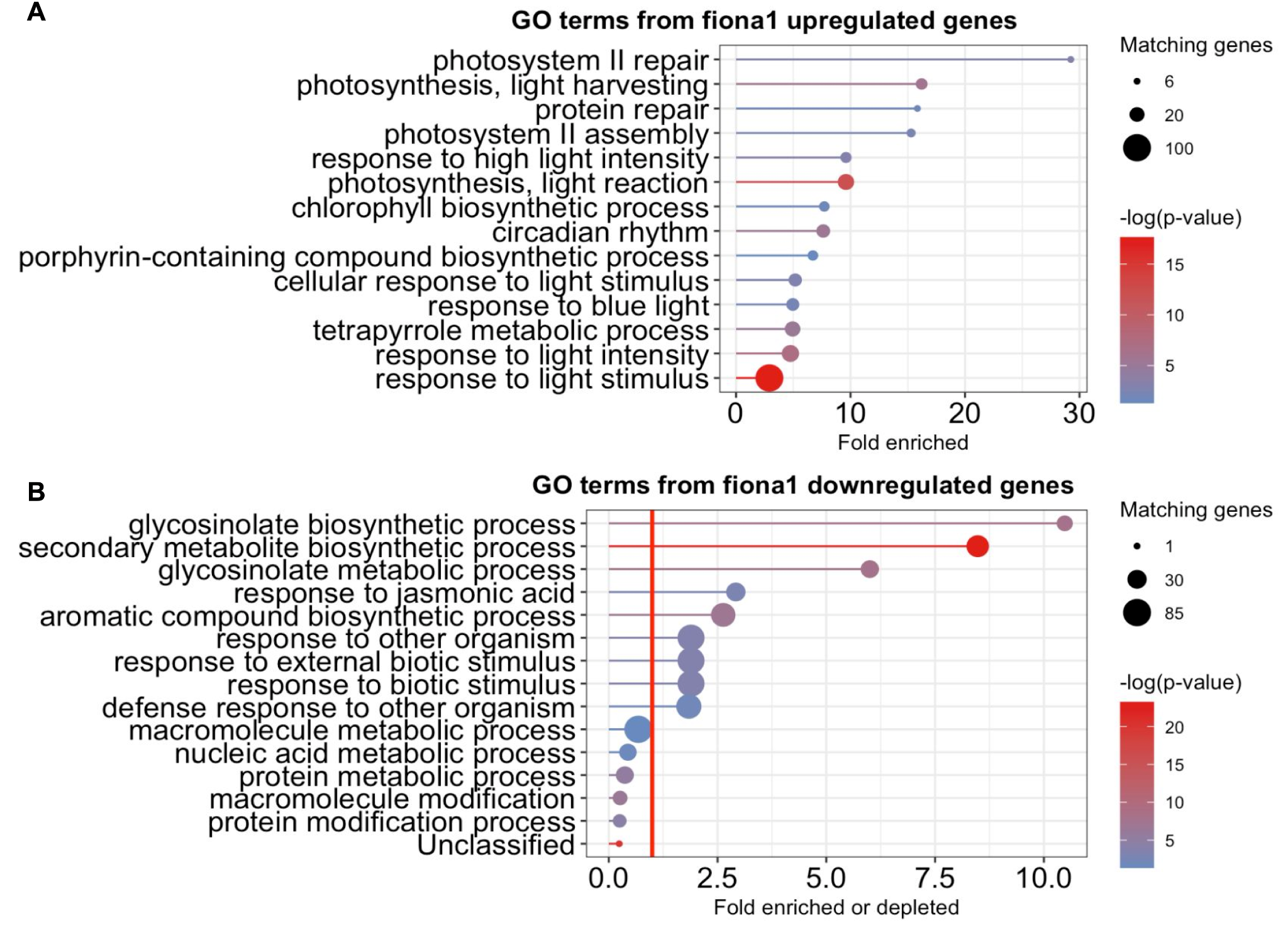


**Supplemental Figure 5: GO analysis of published f*iona1* mutant data reveals many of the same trends identified in our data.** A) Lollipop plot of selected Gene Ontology (GO) terms enriched among *fiona1* upregulated genes. Dot size indicates number of genes, line length indicates fold enrichment, color indicates statistical significance. All GO categories displayed are enriched, the lowest enrichment is 2.91x. B) Lollipop plot of selected GO terms enriched among *fiona1* downregulated genes. Dot size indicates number of genes, line length indicates fold enrichment, color indicates statistical significance. GO categories with the circle to the right of the red vertical line are enriched, those to the left of the line are depleted. Full lists of GO categories and associated data are available in Supplemental Table 9 and Supplemental Table 10 for the upregulated and downregulated gene lists respectively.
