# Supplementary material for: Expression of a mammalian RNA demethylase increases flower number and floral stem branching in Arabidopsis thaliana
Source: Plant Direct. 2024 Aug 21;8(8):e70000. doi: 10.1002/pld3.70000 (PMC11636547; doi:10.1002/pld3.70000)
Supplement: Supplementary file 6 — Data S1 Supporting Information. [file PLD3-8-e70000-s002.pdf]

Plant Direct
Open Access

Expression of a mammalian RNA demethylase increases flower number and floral stem branching in *Arabidopsis thaliana*

Kasey Markel[1,2,3], Lucas Waldburger[3,4,5] , Patrick M. Shih[1,2,3,6,7]*

Decision Letter Round 1:

June 12, 2024

Patrick M Shih
University of California Berkeley
Berkeley, California

RE: Expression of a mammalian RNA demethylase increases flower number and floral stem branching in Arabidopsis thaliana

Dear Dr. Shih:

Thank you for utilizing the transfer function to Plant Direct, which we hope is a convenient option for sharing your work. Based on my evaluation of the manuscript, and informed by the decision summary and reviews from the partner journal, I am pleased to inform you that your manuscript would be suitable for publication with Plant Direct after addressing some concerns with revisions.

Overall, I can readily see this is important work illuminating the effects of human RNA demethylase in plants. On the other hand, I would like to more clearly see in the abstract and discussion which assertions directly follow the evidence and which are new hypotheses that are not yet fully proven. In other words, please make sure conclusions are justified by results, and be transparent when making speculation (which is OK to do in discussion). Also please check the methods to ensure the work is fully reproducible by another lab. The previous reviewers both indicated the work is sound, but especially reviewer 1 indicated major experimental work that would be needed to make the results conclusive. Rather than new experiments, you may state these limitations and how they might be addressed in the future within the discussion section.

Please also address any other reviewer comments from the previous journal, which are included below.

When uploading the revised version of this article, please be sure to include the following:

-A word document that contains your response to the reviewers. You should respond to each reviewer comment and note the changes made to the manuscript. If you do not agree with a reviewer's comment and choose not to make a suggested revision, please explain why. Please try to provide as complete an answer as possible to each reviewer's criticisms.
-A tracked changes document with each change highlighted
- A clean version of the latest version of the manuscript

To upload your revision, please click the link below.
https://plantdirect.msubmit.net/cgi-bin/main.plex?el=A1Lr1hm3A4olv1I1A9ftd6YXq3vhBqJ8qUJwa9yRmgZ

In order to provide as timely a service as possible, we ask that your revision is resubmitted within three months after receipt of this request. If an extension is needed, please send a request, along with a brief explanation, to the editorial office at plantdirect@wiley.com .

Please note that, in addition to publishing reviewer comments, the author's responses to review comments will also be published alongside the final version of the paper. If you would not like the author's responses to be published, please contact the editorial office at plantdirect@wiley.com .

Thank you very much for giving us an opportunity to review your work. I look forward to receiving the next version.

Sincerely,

Larry York

Editor-in-Chief, Plant Direct

---Previous Reviewer comments:

Reviewer #1 (Comments for the Author):

In this manuscript, the authors demonstrate that human FTO overexpression in Arabidopsis can enhance yield traits reminiscent to those reported in rice. They applied RNA-seq, analyzed differential gene expression in Arabidopsis, and proposed how FTO might enhance yield traits. The work is solid. There are a few points the authors need to address before can be accepted by Plant Physiology,

Major:
1) In the manuscript, the authors present the phenotype of OE-FTO plants without confirming FTO expression in transgenic plants. Additional analyses such as western blot and RT-PCR are required to verify FTO expression. To characterize the early bolting phenotype, measuring total leaf number could be added. Additional data elucidating the early flowering phenotype in OE-FTO plants, particularly regarding m6A levels on flowering-related genes such as FT and FLC would be nice.

2) Through RNA-seq analysis the authors suggested that the SAUR gene families are major targets of FTO for increased yield traits in OE-FTO plants. Confirming the expression of SAUR gene families through qPCR analysis is required. m6A-IP-qPCR can be employed to measure m6A level changes on SAUR family genes in Arabidopsis with and without FTO. Future work could involved genetic KD or KO SAUR genes in OE-FTO plants to validate the discovery. But this could be a different work.

Minor:
1) Figure1, "FTO-1, FTO-2" should be "FTO-1, FTO-2".
2) Line 497, "day-old seedlings" should specify the age.
3) Line 173, "FTO" should be "FTO", and many "FTO" should be formatted in the manuscript.

Reviewer #2 (Comments for the Author):

The authors report their phenotypic observations following the overexpression of the human enzyme FTO, a demethylase required for adenosine demethylation (m6A) in mRNA, which is absent in plants. The manuscript also contains a detailed description of the accompanying RNA-seq data. Overexpression of FTO, including the detection of m6A marks and phenotypes, has been described earlier in both monocot and dicot models. Therefore, it is difficult to find significant novelty in the submitted manuscript.
For future authors' consideration: Note it was found that SAUR transcripts likely do not carry m6A marks. Hence, among the strongly misregulated transcripts (both up and down) presented here, I found enriched also those that do not seem to carry m6A.
Other comments:
The bioinformatics analysis of the transcriptomic data is thorough. The conclusions are strong but too speculative.

The validation of expression pattern of the transgene is missing.
Introduction: The link between biomass production in plants and obesity in humans feels inappropriate.

Decision Letter Round 2:

August 6, 2024
Patrick M Shih
University of California Berkeley
Berkeley, California

MSID: 2024-01499-TR1
MS TITLE: Expression of a mammalian RNA demethylase increases flower number and floral stem branching in Arabidopsis thaliana

Dear Dr. Patrick M Shih:

Thank you for utilizing our transfer feature, which I hope was a convenient option to share your work. I am pleased to inform you that your manuscript "Expression of a mammalian RNA demethylase increases flower number and floral stem branching in Arabidopsis thaliana" has been accepted for publication in Plant Direct.

Your article will appear online in the next available issue of Plant Direct. To ensure your article gets published as quickly as possible, please pay attention to the steps detailed below. We have found that most of the delays happen at this stage, especially at the payment stage, so please respond as quickly as possible when prompted.

License Agreement: Once your article has been accepted it will move to Production and undergo admin and file checking - you may receive an email with any queries we have at this stage. When all required items are received by the publisher and queries resolved, the corresponding author will receive an email from Wiley's Author Services system which will ask them to log on at https://authorservices.wiley.com/bauthor and will present them with the appropriate license for completion. Your article cannot be published until both the signed license agreement and payment of the article fee have been received.

Payment of the Open Access Article Publication Fee: All articles published in Plant Direct are fully open access: immediately and freely available to read, download and share. Plant Direct

charges a publication fee to cover the publication costs. The corresponding author for this manuscript should have already received a quote from the payments team (cs-openaccess@wiley.com) with the estimated article publication fee; please email cs-openaccess@wiley.com if this has not been received. The corresponding author should log on to the Wiley Author Services site, where the publication fee can be paid by credit card or an invoice. Pro Forma can also be requested. Payment of the publication charge must be received before the article will be published online.

Proofs: You will have the opportunity to look over your paper once more when you receive the author proofs for your article. The proofs will be with you in approximately two weeks. Please note that, in addition to publishing reviewer comments, the author's responses to review comments will also be published alongside the final version of the paper. If you would not like the author's responses to be published, please contact the editorial office at plantdirect@wiley.com .

Promotion of your article: You can help your research get the attention it deserves! Check out Wiley's free Promotion Guide for best-practice recommendations for promoting your work at www.wileyauthors.com/eeo/guide. And learn more about Wiley Editing Services which offers professional video, design, and writing services to create shareable video abstracts, infographics, conference posters, lay summaries, and research news stories for your research at www.wileyauthors.com/eeo/promotion.

Thank you again for your contribution to Plant Direct. If you have any questions, feel free to contact the editorial office at plantdirect@wiley.com .

Sincerely,

Larry York

Editor, Plant Direct

Author's Response to Reviewer:

We thank both reviewers for comments which have strengthened our manuscript.

Reviewer #1 (Comments for the Author):

In this manuscript, the authors demonstrate that human FTO overexpression in Arabidopsis can enhance yield traits reminiscent to those reported in rice. They applied RNA-seq, analyzed differential gene expression in Arabidopsis, and proposed how FTO might enhance yield traits. The work is solid.

Thank you for the kind summary.

There are a few points the authors need to address before can be accepted by Plant Physiology,

Major:

1) In the manuscript, the authors present the phenotype of OE-FTO plants without confirming FTO expression in transgenic plants. Additional analyses such as western blot and RT-PCR are required to verify FTO expression. To characterize the early bolting phenotype, measuring total leaf number could be added. Additional data elucidating the early flowering phenotype in OE-FTO plants, particularly regarding m6A levels on flowering-related genes such as FT and FLC would be nice.

Expression of FTO was confirmed through the RNAseq data for three of our FTO lines, sequences mapping to FTO are present in the raw reads (as can be verified through the full read data available online).

That said, because expression of FTO in our FTO-transgenic lines is a key supposition for our analysis, we have now also performed RTqPCR to confirm expression of FTO in all lines analyzed in the paper. This is now included as Supplemental Figure 1 and other supplemental figure numbers have been adjusted accordingly. We believe that RTqPCR is sufficient to establish expression of the transgene, and therefore decline to perform Western blots.

We considered experiments to quantify m^{6}A levels in specific genes of interest, but given the highly specialized nature of such experiments and the urgency to report replication findings, we have opted to let those experiments wait for future research - we believe that we are already reporting a sufficiently wide range of results for one paper.

2) Through RNA-seq analysis the authors suggested that the SAUR gene families are major targets of FTO for increased yield traits in OE-FTO plants. Confirming the expression of SAUR gene families through qPCR analysis is required. m6A-IP-qPCR can be employed to measure m6A level changes on SAUR family genes in Arabidopsis with and without FTO. Future work could involved genetic KD or KO SAUR genes in OE-FTO plants to validate the discovery. But this could be a different work.

We identified the SAUR overrepresentation through RNA-seq results performed with 6 biological replicates per line, using the even stricter cutoff of only the overlap in the top-100 most upregulated genes in multiple independent lines. We therefore are fairly confident in this finding even in the absence of RTqPCR verification, and decline to perform additional assays to confirm the expression level of the four SAUR genes we identified as highly overexpressed.

We agree that m^{6}A-IP-qPCR would be an interesting analysis to run, but don't believe it is critical for our conclusions. Notably, we don't claim that the upregulation of SAUR genes is dependant on changes to the m^{6}A state of those genes themselves - we find it most likely that FTO is causing other regulatory changes upstream of SAUR regulation, and a negative result for m^{6}A-IP-qPCR on the SAUR genes wouldn't change the fact that growth-associated SAUR genes are highly upregulated in our FTO lines, presenting a plausible hypothesis for a mechanism behind FTO's growth-enhancing phenotype.

We agree that KD or KO of SAUR genes in FTO lines is an interesting future direction and hope that such experiments will be performed.

Minor:

1) Figure1, "FTO-1, FTO-2" should be "FTO-1, FTO-2".

The proposed error and proposed correction here are identical. We believe the labels in Figure 1 are correct.

2) Line 497, "day-old seedlings" should specify the age.

Thank you, this somehow got lost during editing. The seedlings were 7 days old, the manuscript has now been corrected.

3) Line 173, "FTO" should be "FTO", and many "FTO" should be formatted in the manuscript.

Here again the claimed error and the suggested solution are identical. We believe that FTO (capital letters, no italics, no bold, lower case and italics in the few cases when we discuss *fto* mutants) is correctly written in this manuscript, but welcome further clarification if there is something we're missing here.

Reviewer #2 (Comments for the Author):

The authors report their phenotypic observations following the overexpression of the human enzyme FTO, a demethylase required for adenosine demethylation (m6A) in mRNA, which is absent in plants. The manuscript also contains a detailed description of the accompanying RNA-seq data.

Thank you for the kind summary.

Overexpression of FTO, including the detection of m6A marks and phenotypes, has been described earlier in both monocot and dicot models. Therefore, it is difficult to find significant novelty in the submitted manuscript.

We believe the most valuable aspect of our manuscript is that it's an independent replication of an extremely surprising finding that (rightfully in our opinion) generated both a lot of excitement and a lot of skepticism in the plant biology community.

However, we believe our manuscript also includes many novel elements - we perform experiments in a new species which has never been characterized with FTO overexpression before, we perform a much more detailed RNA-seq analysis than any existing for FTO-overexpressing plants, and come up with many interesting plausible hypotheses for some of the mechanisms behind FTO's growth-enhancing phenotype. Furthermore, we identify several patterns not previously found in FTO plants such as the growth-defense tradeoff in both our and in the data from the only previous FTO-plant paper, and find several somewhat surprising sequence-level differences between the FTO-upregulated and downregulated gene sets.

For future authors' consideration: Note it was found that SAUR transcripts likely do not carry m6A marks. Hence, among the strongly misregulated transcripts (both up and down) presented here, I found enriched also those that do not seem to carry m6A.

Interesting! We don't claim that the upregulation of SAUR genes is due to any change in the m^6A status of the SAUR genes themselves, we simply note that these growth-associated genes are highly overrepresented among our top upregulated genes. Your claim supports our hunch that the upregulation of SAUR genes is due to upstream regulatory action.

Other comments:

The bioinformatics analysis of the transcriptomic data is thorough. The conclusions are strong but too speculative.

Thank you for the kind description. We agree that our manuscript, as the first independent report of an extremely surprising and novel finding, contains some speculation. We believe this is valuable at this early stage in the understanding of FTO's function in plants.

We have changed some of our language to make it more clear when we are making conclusions solidly grounded by our data and when we are speculating.

The validation of expression pattern of the transgene is missing.

We have performed RTqPCR for FTO expression and added that data as Supplemental Figure 1.

Introduction: The link between biomass production in plants and obesity in humans feels inappropriate.

FTO is best known as an obesity-related gene, and we thought it useful and appropriate to provide the background. We don’t claim that FTO’s biomass-enhancing mechanisms in mammalian cells are the same as biomass-enhancing mechanisms in plants, though we also don’t reject that hypothesis.
